# Structural Domain Analysis of Heparin and Heparan Sulfate Combined With Label-Free Quantitative Proteomics to Elucidate Their Functional Diversity in Liver Cancer and APAP-Induced Liver Injury

**DOI:** 10.1016/j.mcpro.2025.101090

**Published:** 2025-10-14

**Authors:** Zizhe An, Qingqing Chen, Changkai Bu, Lexin Chen, Deling Shi, Bin Zhang, Lan Jin, Lianli Chi

**Affiliations:** 1 National Glycoengineering Research Center, Shandong University, Qingdao, Shandong Province, P.R. China; 2 School of Pharmaceutical Sciences, Shandong University of Traditional Chinese Medicine, Jinan, P.R. China; 3 Shandong Key Laboratory of Carbohydrate and Carbohydrate-conjugate Drugs, Shandong University, Qingdao, Shandong Province, P.R. China

**Keywords:** heparin, heparan sulfate, structural analysis, liver cancer, liver injury

## Abstract

The precise structure of glycosaminoglycans is critical for their bioactivity and the development of glycopharmaceuticals. Herein, cellular and animal experiments were conducted to assess the differences in the activities of heparin (HP) and heparan sulfate (HS) against liver cancer and drug-induced liver injury. Label-free quantitative proteomics, bioinformatics, biolayer interferometry, and immunohistochemical analyses were used to determine key proteins with differential expression. As a result, HP demonstrated superior antiliver cancer activity compared with HS, whereas HS exhibited strong potential in resisting acetaminophen-induced liver injury. DIRAS family GTPase 2 (DIRAS2) was identified as a key HS-binding protein that was strongly associated with cell proliferation, and its expression levels in cells and tissues showed opposite trends following HP and HS administration. HP significantly reduced the abundance of DIRAS2 in the tumor tissue, thereby inhibiting tumor cell proliferation, whereas HS promoted proliferation by increasing DIRAS2 expression. Cluster sequencing revealed that consecutive GlcNS6S-IdoA2S domains in HP and IdoA2S-GlcNS6S, GlcA-GlcNS6S, and IdoA-GlcNAc domains in HS were required for affinity binding within the decasaccharide region. Molecular docking suggested that differences in the binding modes of HP and HS chains to DIRAS2 underlie their functional diversity. These findings indicate that HP and HS oligosaccharides with well-defined structures may serve as potential therapeutic agents for liver-related diseases.

## Highlights

• HP and HS were found to have opposite effects on cell proliferation in liver diseases.
• DIRAS2 was identified as a key HSBP that affects liver cell proliferation.
• Different domains in HP and HS were the factor that affected their bioactivity.

## Graphical Abstract

**Figure:**
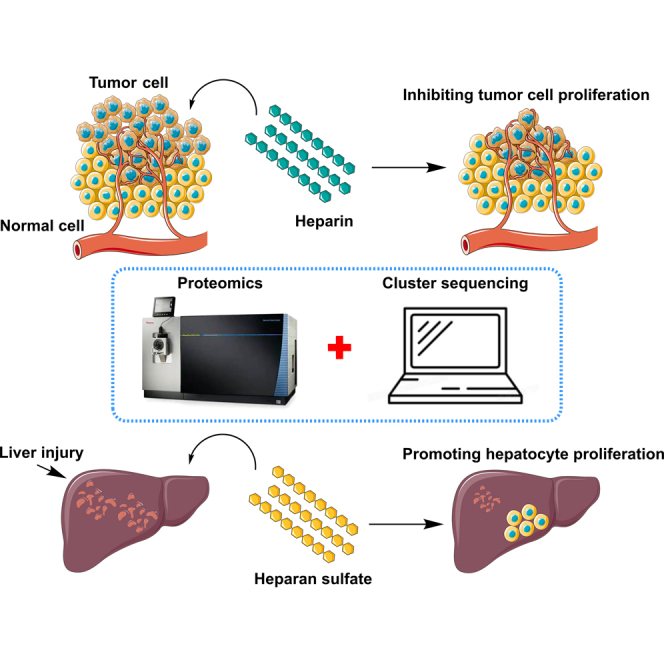

## In Brief

Liver cancer and DILI are at a high level of incidence rate worldwide, and the investigation of less toxic alternative drugs is urgently needed, of which HP and HS have been of great interest to clinical applicators. Both HP and HS perform their promoting or inhibiting cell proliferation activities in liver diseases by interacting with specific proteins based on their bioactive domains. However, the precise structure of these active sequences and the molecular mechanisms of their interaction with specific proteins still require further investigation. In our present study, nonlabeled quantitative proteomics, bioinformatics, BLI, and immunohistochemical analysis were used to identify the differential proteins involved in cell proliferation under HP and HS administration. Cluster sequencing technique was used to analyze the sequences of HP/HS chains that interacted with the targeted protein. Our study demonstrated that HP/HS oligosaccharide with well-defined structures could be potentially developed as antiliver disease drugs.

The liver, the largest digestive gland in the human body, is the primary organ responsible for material and energy metabolism. Consequently, liver diseases, including hepatitis (1), cirrhosis (2), liver abscess (3), and primary liver cancer (4), pose serious threats to human health. Liver cancer and drug-induced liver injury (DILI), in particular, have a high incidence rate globally (5, 6). Primary liver cancer is mainly characterized by abnormal proliferation of hepatocytes (5), and therapeutic strategies often focus on inhibiting tumor cell proliferation and angiogenesis. Similarly, DILI is characterized by abnormal necrosis and inflammation of hepatocytes caused by toxic substances (6), and clinical treatment primarily aims to suppress inflammation while promoting tissue repair and regeneration. Therefore, direct and effective intervention in cell proliferation during liver cancer and liver injury constitutes a key therapeutic strategy. Pharmacological treatments for liver cancer generally include targeted drugs, immunotherapies, and chemotherapies. For example, sorafenib is a representative drug that inhibits angiogenesis in the tumor microenvironment (7). Conversely, pharmacological treatment of DILI is mainly based on replenishing intracellular levels of reduced glutathione and *N*-acetylcysteine (NAC) (8), both of which exert strong antioxidant effects. However, the major drawbacks of these compounds are their severe side effects and the development of drug resistance. Thus, the search for less toxic alternative therapies is critical.

In the development of alternative drugs with fewer side effects, sulfated polysaccharides as natural molecules with prominent antioxidant (9), anti-inflammatory (10), and antiangiogenic activities (11) have garnered considerable clinical interest. Representative examples include heparin (HP) and heparan sulfate (HS). HP (∼2.3 sulfate groups per disaccharide) and HS (∼0.8 sulfate groups per disaccharide) both belong to the glycosaminoglycan (GAG) family and share the same basic repeating unit, (GlcA/IdoAβ1→4GlcNAcα1→4)_n_. In these chains, the 3-, 6-, and *N*-positions of glucosamine residues or the 2-position of uronic acid can be substituted with the sulfate groups, and HP can thus be regarded as a highly sulfated form of HS (12). Unlike HP, which is synthesized and stored in mast cells, HS is typically bound to serine-rich core proteins to form HS proteoglycans (HSPGs) that localize to the cell surface. HP had been reported to exert inhibitory effects on tumor angiogenesis as well as on tumor cell proliferation, invasion, and migration (13). In addition, HP could inhibit cell migration by downregulating the expression of heparanase (14). Likewise, HS could act as an anti-inflammatory agent that protected against acetaminophen (APAP)-induced acute liver failure by interacting with the high mobility group box 1 (HMGB1) protein (15). Previous studies had demonstrated that HSs and HSPGs played important regulatory roles in hepatocyte proliferation (16, 17). For example, purified HSPG (syndecan-1), HS, or synthetic HP analogs containing 2-*O*-sulfate groups had been shown to enhance AKT signaling and inhibit GSK-3β-induced hepatocyte apoptosis, thereby protecting Sdc1^−/−^ mice from APAP-induced liver injury (16). Compared with NAC, the therapeutic effect of HS was significantly prolonged. Moreover, HSPGs on the surface of tumor cells contain bioactive sequences that influence tumor cell growth and metastasis. Importantly, these sequences include both growth-promoting and growth-inhibiting domains, and the dynamic balance among these distinct polysaccharide motifs was demonstrated to regulate intracellular signaling pathways in tumor cells (17). However, the precise structural sequences of these bioactive domains in HS and HSPGs remain poorly characterized.

It is recognized that both HP and HS perform their biological activities by interacting with specific proteins through defined bioactive domains. Classic examples demonstrated that these interactions depend on specific sequences rather than occurring in a disordered manner. For instance, the interaction of HP with antithrombin requires a specific 3-*O*-sulfated, but not fully sulfated, pentasaccharide sequence [-GlcNS6S/GlcNAc6S-GlcA-GlcNS3S/GlcNS3S6S-IdoA2S-GlcNS6S-], which is generally absent in HS chains (18). Likewise, a synthetic HS octadecasaccharide with the sequence [GlcNS-GlcA-(GlcNS-IdoA2S)_7_-GlcNS-GlcA], which exhibited low anticoagulant activity, had been shown to function as a targeted agent that protected against sepsis by directly binding with cytotoxic histone H3 (19) and as an anti-inflammatory agent that protected against APAP-induced acute liver failure by interacting with HMGB1, a key mediator of inflammation (15). These findings suggested that HP and HS, through their bioactive domains, could specifically interact with target proteins. However, their functional variability in disease-specific contexts, particularly with respect to refined structural features such as sulfation patterns and precise saccharide sequences, remains poorly understood. Moreover, the structural heterogeneity of HP and HS chains presents significant practical challenges for clinical applications.

In the present study, label-free quantitative proteomics of HepG2 cellular models, bioinformatics analysis, immunohistochemical validation in animal models, and GAG cluster sequencing technologies were employed to elucidate the functional differentiation and molecular mechanisms of HP and HS in liver cancer and APAP-induced liver injury. HP was found to have greater therapeutic potential in liver cancer, whereas HS showed promise in the treatment of APAP-induced liver injury. This study clarified the refined structure–function relationships of HP and HS in liver diseases and lays the groundwork for developing targeted HS-like carbohydrate-based therapies.

## Experimental Procedures

### Materials and Chemicals

HP (molecular weight [Mw ∼ 16 kDa, purity ≥99%) was obtained from the US Pharmacopeia. HS (Mw ∼ 33 kDa, purity ≥95%) was purchased from Asnail. Heparinase I, II, and III (specific activity: 400 IU/mg, 15 IU/mg, and 200 IU/mg) and disaccharide standards (ΔIS, ΔIIS, ΔIIIS, ΔIVS, ΔIA, ΔIIA, ΔIIIA, ΔIVA, and ΔIP) were purchased from Asnail. Water, acetonitrile of LC–MS grade, cell lysis solution, and bicinchoninic acid (BCA) protein quantification kit were purchased from ThermoFisher Scientific, and ammonium acetate/formate of LC–MS grade, iodoacetamide, and dithiothreitol were purchased from Sigma–Aldrich. Amine PEG_3_–Biotin (purity ≥97%) was purchased from ThermoFisher Scientific. Sequencing-grade trypsin was purchased from Promega. Ziptip C_18_ column was purchased from Merck. Deuterium oxide (D_2_O, 99.96% atom D) was purchased from Tenglongweibo. Sensor SA chip and cyanogen bromide–activated Sepharose 4B resin were purchased from Cytiva. HepG2 cells were purchased from Boster. BALB/C and C57BL/6J mice were obtained from Vital River. PBS solution, 4% polyformaldehyde, and saline were obtained from Procell. The antibody of HMGB1, Ki-67, and DIRAS family GTPase 2 (DIRAS2) was purchased from Abcam. DIRAS2 (NM_017594, human recombinant protein purified from human embryonic kidney 293T) was purchased from Abmart. Bovine serum albumin was purchased from Solabio. DIRAS2 polyclonal antibody, GAPDH monoclonal antibody, horseradish peroxidase (HRP)–conjugated AffiniPure goat anti-rabbit IgG (H + L), HRP-conjugated AffiniPure goat anti-Mouse IgG (H + L), and other chemicals and reagents for Western blotting were purchased from Proteintech. T-PER protein extraction reagent was purchased from ThermoFisher Scientific. All other chemicals and reagents were of analytical grade (purity ≥99%) and obtained from Sinopharm Chemical Reagent Co, Ltd.

### Multiple Reaction Monitoring Analysis of HP and HS

The disaccharide building block of HP and HS was performed by the reported LC–MS/MS multiple reaction monitoring (MRM) method (20). Enzymatic digestion was performed by incubating HP and HS (30 μg, respectively) with 10 mIU heparinase I, II, and III at 37 °C for 48 h. Then the solution was lyophilized. AMAC solution (10 μl 0.1 mol/l) was added into the disaccharide product and dark reacted at 25 °C for 10 min. Then, 10 μl of 1 mol/l NaBH_3_CN was added into the solution and reacted at 45 °C for 3 h. Sample diluent (dimethyl sulfoxide:CH_3_COOH:H_2_O = 17:3:20) was added to dilute the sample to a final concentration at 1 μg/μl. Quantitative analysis of eight disaccharides was performed on UHPLC-Triple Quad 5500^+^. The C_18_ column was Kinetex EVO C18 (2.1 × 150 mm, 2.6 μm; Phenomenex). Eight HP disaccharide standards (ΔIS, ΔIIS, ΔIIIS, ΔIVS, ΔIA, ΔIIA, ΔIIIA, and ΔIVA) were used as external standards, and ΔIP was used as an internal standard for data calibration and quantification. Mobile phase A was 50 mM NH_4_Ac, and mobile phase B was methanol. The gradient was 0 to 10 min, 5% to 45% B; 10 to 12 min, 45% to 100% B; and 12 to 16 min, 100% B. The flow rate was 0.2 ml/min, and the column temperature was set at 45 °C. The parameters of the MS and MS/MS were consistent with those reported in the reference and listed in Table S1.

### 2D NMR–Heteronuclear Single Quantum Coherence Analysis of HP and HS

The HP and HS samples (30 mg, respectively) were dissolved in 550 μl of 99.96% D_2_O and then lyophilized three times for deuterium exchange and finally dissolved in 500 μl of 99.96% D_2_O for NMR analysis (21). One-dimensional (^1^H-NMR and ^13^C-NMR) and two-dimensional spectra (2D NMR–heteronuclear single quantum coherence [HSQC]) were performed at 298 K on a Bruker AVANCE NEO 600 MHz with TopSpin software.

### Effect of HP and HS on Cell Proliferation

The HepG2 cell was incubated with Dulbecco's modified Eagle's medium containing 10% fetal bovine serum at 37 °C and 5% CO_2_ concentration. The drug treatment was initiated after the cell had been spread in 96-well plates for 24 h, and the cell fusion rate was about 80%. The experiment was divided into control, HP, and HS groups with six replications. The control group was treated with an equal amount of saline, and the HP and HS groups were administered a concentration of 50, 100, 200, 300, 400, 500, and 600 μg/ml. The cell proliferation rate of HP or HS treated by heparinase I or heparinase III was also carried out according to the aforementioned steps, and the administered concentrations of HP and HS were both 300 μg/ml. The common approach of 3-(4,5-dimethyl-2-thiazolyl)-2,5-diphenyltetrazolium bromide method was used to determine the cell proliferation rate.

### Experimental Design and Statistical Rationale

For label-free quantitative proteomics of the HepG2 cellular model, the protein extraction and proteomics methods were performed according to the method reported before with minor modification (22). The HepG2 cell was incubated with complete medium for 24 h, then the aforementioned complete medium was replaced with the complete medium containing HP/HS (300 μg/ml) and incubated for 24 h. After digesting, centrifuging, and washing the collected cells by PBS solution, 300 μl lysis buffer containing protease inhibitor and phosphatase inhibitor were added, and the cells were lysed on ice for 20 min. The supernatant was then collected and quantified by the BCA protein quantification kit, followed by desalination using a 2 kDa ultrafiltration membrane. Then 300 μl of 50 mmol/l dithiothreitol solution was added into 200 μg protein and reacted at 37 °C for 4 h. After ultrafiltration to fully remove the dithiothreitol, 300 μl of 50 mmol/l iodoacetamide solution was added and reacted at 25 °C for 1 h. After ultrafiltration to remove the iodoacetamide, 100 μl of 20 mmol/l NH_4_HCO_3_ solution and 10 μg of sequencing-grade trypsin was added and digested at 37 °C for 12 h. Then the peptides were collected by centrifuging at 3000 rpm. After that, 50 μg of peptide mixture were dissolved in 150 μl of ammonia (pH = 10) and then separated by a C_18_ column (Merck), which was sequentially eluted by 200 μl of acetonitrile solution of different concentrations (6%, 9%, 12%, 15%, 18%, 21%, 25%, 30%, 35%, and 50%, respectively). The eluates were combined, and the peptide samples were obtained after freeze–drying.

A 1 μl peptide sample (1 mg/ml) was subjected to electrospray ionization high-resolution mass spectrometry (ESI–HRMS) for proteomics analysis. Reversed-phase separation of peptide was performed on a capillary C_18_ column on an Easy nLC1200 system (ThermoFisher Scientific). Eluent A was 0.1% formic acid solution, and eluent B was 0.1% formic acid prepared in 80% acetonitrile. A gradient of 2% to 10% B (0–4 min), 10% to 28% B (4–44 min), 28% to 38% B (44–55 min), 38% to 55% B (55–60 min), 55% to 95% B (60–65 min), and 95% B (65–75 min) was used at a flow rate of 300 nl/min for peptide separation. The ESI–HRMS was carried out on a Thermo Orbitrap Fusion Lumos mass spectrometer (ThermoFisher Scientific). The electrospray interface was set in positive ion mode (scan range, *m/z* 200–2000) at a resolution of 60,000 with a capillary temperature of 320 °C, a spray voltage of 3.80 kV, and an S-lens RF level of 50. The eluted peptides were sprayed into a mass spectrometer *via* a nanospray ion source. The Nano Orbitrap Fusion Lumos Tribrid MS with Advanced Peak Determination was run in cycle time data-dependent acquisition mode with Xcalibur 4.2.47 software (Thermo Scientific), and the time between master scans was 3 s. The precursor ion intensity threshold greater than 4.0e5 in the quadrupole was selected for MS/MS fragmentation analysis at a normalized collision energy of 30% in Orbitrap detector with a resolution of 15,000. In order to avoid repetitively selecting peptides, the dynamic exclusion duration was 25 s.

For experimental design, data analysis, and statistical rationale of proteomics analysis, cellular proteomics of six biological replicates and three technical replicates were performed per group (control, HP, and HS), except for differences in drug treatment, all subsequent processing steps for protein processing were identical. Data analysis was performed on Thermo Proteome Discoverer software (version 2.3; Thermo Fisher Scientific) with the Sequest HT search engine. Sample groups were set as C (C1, C2, C3, each containing six files), HP (HP1, HP2, HP3, each containing six files), and HS (HS1, HS2, HS3, each containing six files). Generated ratios were set as C–HP, C–HS, and HP–HS. The data search conditions were set as follows: protein database (*Homo sapiens*, downloaded date August 04, 2021 from UniProt, 177,092 entries), trypsin, up to two missed cleavages, peptide length of 4 to 144, mass tolerance of 10 ppm and fragment mass tolerance of 0.02 Da, variable modification as oxidative modification and acetylation modification of *N*-terminal methionine, and fixed modification as iodoacetamide modification of cysteine, and false discovery rate for peptide and protein both as 0.01 (high confidence) or 0.05 (medium confidence). In the workflow, label-free quantification was according to precursor intensity (peptide to use, unique + razor), and the normalization mode was set as total peptide amount. After quantification, we defined proteins with fold change >2, *p* < 0.05 as differentially expressed proteins. The original documents detailing the search method and result could be found on the ProteomeXchange Consortium (https://proteomecentral.proteomexchange.org) under dataset identifier PXD064406.

### Bioinformatic Analysis

Gene Ontology (GO) enrichment analysis of differentially expressed proteins and Kyoto Encyclopedia of Genes and Genomes pathway enrichment analysis and visualization were performed on the website tool (https://www.bioinformatics.com.cn) for data analysis and visualization, with adjusted *p* < 0.05 set as the cutoff criteria. The protein–protein interaction network was constructed from the STRING database and visualized by Cytoscape software (V3.10.0, Institute of Systems Biology).

### Western Blotting of Targeted Protein

To HepG2 cells of control, HP, and HS groups in the cell culture flask were added 3 ml PBS with gentle shaking for 1 min, repeating three times. Then, the cells were scraped, and 400 μl of T-PER protein extraction reagent containing 1% protease inhibitor and phosphatase inhibitor was added to extract the total protein. The total protein concentration was determined by the BCA protein assay kit. Each sample was mixed with 4× loading buffer and boiled for 5 min. Electrophoresis parameters were as follows; 12.5% SDS-PAGE gel, 20 μg sample, 80 mA for 20 min, and 120 mA constant current for 60 min. Protein transferring to polyvinylidene fluoride membrane parameters was as follows; electrolysis for 100 min at 4 °C under 110 V constant current condition. After blocking with 5% skim dry milk, the primary antibody and secondary antibody incubation were performed sequentially. Three biological replicates were performed. The VILBER Fusion FX Imaging System was used for imaging, and ImageJ (https://imagej.net/ij/index.html) was used for data processing.

### Binding Affinity Measurement of HP–HS With Targeted Protein by Biolayer Interferometry

The biolayer interferometry (BLI) experiment was used for characterizing the interaction between HP–HS and targeted protein according to the reported method (23). BLI experiments were performed on an Octet RED96 system (Sartorius) with ForteBio Data Analysis 10.0 software. Biotinylated HP/HS was prepared by coreaction of HP/HS (2 mg, respectively), amine PEG_3_–Biotin (2 mg), and 20 mg NaCNBH_3_ at 70 °C for 48 h, followed by desalting by gel permeation chromatography (GPC) using the Superdex Peptide 10/300 GL column (9 μm, 10 × 300 mm; Cytiva) using ultrapure water as the mobile phase and then lyophilized for 24 h. The protein master mix was diluted to 1, 0.5, 0.25, 0.125, 0.0625, and 0.0313 μM with PBST (PBS containing 0.02% Tween-20) solution. The procedure was performed as follows: sensor prewetting: 600 s, PBST; baseline: 60 s, PBST; loading: 180 s, biotinylated HP–HS; baseline2: 180 s, PBST; association: 300 s, protein master mix; dissociation: 300 s, PBST; temperature: 30 °C; and acquisition rate: standard kinetics (5.0 Hz, averaging by 20), fitting and data analysis with a 1:1 Langmuir model. Bovine serum albumin was used as a negative control for HP/HS–protein interaction.

### Establishment of Transplanted Tumor, APAP-Induced Liver Injury, and Drug Administration Models

For the transplanted tumor model, 8-week-old BALB/c mice were adaptedly fed for 1 week and randomly divided into four groups (control group, sorafenib group, HP group, and HS group). HepG2 cells were subcutaneously injected into the forelimb armpit area of mice at a concentration of 1 × 10^6^ cells/pc. After tumor formation, the drugs of 30 mg/kg sorafenib (oral), 50 mg/kg HP (subcutaneous), and 50 mg/kg HS (subcutaneous) were delivered to each group for 5 weeks. The mice were regularly checked, and the tumor diameters were measured every day. At the end of the experiment, the mice were dissected, and the tumor tissues were obtained for subsequent studies. For APAP-induced liver injury model, 8-week-old C57BL/6J mice were adaptedly fed for 1 week and randomly divided into five groups (control group, model group, NAC group, HP group, and HS group). An acute liver injury model was established by intraperitoneal injection of APAP at a concentration of 450 mg/kg. The treatment was administered at 30 min after modeling (300 mg/kg NAC, 100 mg/kg HP, and 100 mg/kg HS, every 12 h once for 72 h) in each group. At the end of the experiment, the blood was taken from the mice, they were dissected, and the liver tissues were obtained for subsequent studies. All animal experiments were approved by the Institutional Animal Care and Use Committee of the Scientific Investigation Committee of Shandong University, and the license number was SYDWLL-2023-075.

### Immunocytochemistry Analysis of Tumor and Liver Tissue

The obtained tissues of tumor and liver were fixed with 4% paraformaldehyde, after which paraffin-embedded sections were prepared. The sections were sequentially soaked in xylene, ethanol, distilled water, and citric acid buffer and then washed with PBS solution. The sections were then incubated with 5% goat serum for 30 min, and the targeted primary antibody and HRP goat anti-rabbit IgG were added onto the sections sequentially. After each incubation, the sections were washed with PBS solution. Finally, 3,3′-diaminobenzidine solution was added onto the sections and incubated at 25 °C for 10 min to reveal color. After the staining was terminated, the nucleus was stained with hematoxylin, and then the sections were dehydrated with alcohol, sealed, observed under the optical microscope with scanning function, and photographed.

### Preparation of Low Molecular Weight HP and Low Molecular Weight HS

HP and HS were depolymerized to oligosaccharides (named as low molecular weight HP [LMW-HP] and low molecular weight HS [LMW-HS]) using the method of partial enzymatic digestion by heparinase I and heparinase III, respectively (24). HP and HS (10 mg) were dissolved into 300 ml of enzymolysis buffer (containing 0.01 mol/l calcium acetate and 0.1 mol/l sodium acetate), and 12.5 μl of 0.4 IU/ml heparinase I/III was added into HP–HS solution for 8 h. Then the major disaccharide product was removed by GPC using a Superdex Peptide 10/300 GL column (9 μm, 10 × 300 mm; Cytiva). The mobile phase of GPC was 0.2 mol/l ammonium hydrogen carbonate, and the flow rate was 0.4 ml/min.

### Affinity Chromatography by Targeted Protein-Sepharose 4B

The affinity chromatography of targeted protein-Sepharose 4B was performed using an optimized procedure reported by Bisio *et al*. (25). The targeted protein was immobilized onto the cyanogen bromide–activated Sepharose 4B resin for 12 h at 4 °C. LMW-HP or LMW-HS was loaded into the affinity chromatography column and eluted with 10 mmol/l Tris–HCl (pH = 7.4), followed by low ionic strength buffer (0.15 mol/l NaCl in 10 mmol/l Tris–HCl, pH = 7.4) and high ionic strength buffer (2.0 mol/l NaCl in 10 mmol/l Tris–HCl, pH = 7.4). The high-affinity fraction eluted at the higher ionic strength buffer was collected, then the fraction eluted at the lower ionic strength buffer was reloaded to the affinity chromatography column and repeated three times for collecting all high-affinity fractions. Afterward, the high-affinity fraction was desalted using a G10 column (40–120 μm, 10 × 400 mm; Elite), followed by freeze–drying. The mobile phase of G10 desalination was ultrapure water, and the flow rate was 1.0 ml/min.

### Characterizing LMW-HP–HS Binding Domain by High-Performance Size-Exclusion Chromatography and Hydrophilic Interaction Liquid Chromatography–ESI–HRMS

High-performance size-exclusion chromatography separations of LMW-HP–HS and their high-affinity fractions were performed on two serially connected Waters ACQUITY UPLC Protein BEH SEC columns (125 Å, 1.7 μm, 4.6 mm × 150 mm and 4.6 mm × 300 mm) on a Vanquish UPLC system (ThermoFisher Scientific) at a flow rate of 0.075 ml/min, respectively, according to the reported method (26). The mobile phase was 50 mmol/l ammonium formate prepared in 20% (v/v) methanol.

A 10 μl sample (2 mg/ml) of LMW-HP–HS and their high-affinity fractions were subjected to hydrophilic interaction liquid chromatography (HILIC)–ESI–HRMS for chain mapping analysis according to the reported method with minor modification (26). HILIC separation of LMW-HP–HS and their high-affinity fractions were performed on a Luna HILIC column (2.0 × 150 mm, 3 μm; Phenomenex) on a Vanquish UPLC system (ThermoFisher Scientific). Eluent A was 5 mmol/l ammonium acetate, and eluent B was 98% acetonitrile with 5 mmol/l ammonium acetate. A linear gradient of 95% to 65% B from 0 to 45 min at a flow rate of 150 μl/min was used for separation. The ESI–HRMS was performed on a Thermo LTQ-Orbitrap XL mass spectrometer (Thermo Fisher Scientific). The electrospray interface was set in a negative ion mode (scan range, *m/z* 400–1500) at a resolution of 60,000 with a capillary temperature of 275 °C, a spray voltage of 4.20 kV, an S-lens RF level of 50, and a sheath gas flow rate at 20 arb. Data analysis was performed on Thermo Xcalibur 3.0 software.

### Cluster Sequencing of LMW-HP–HS Binding Domain by Seq-GAG Software

Cluster sequencing of LMW-HP/HS binding domain was performed according to the reported method (26). The exhaustive enzymatic digestion, nitrous acid degradation of high-affinity LMW-HP/HS, and their ESI–HRMS analysis was carried out following the procedure exactly as reported (24). Seq-GAG software was established and optimized by our group with the Python language (version 2.7.13) and used for cluster sequencing the LMW-HP/HS binding domain, which was validated by previous studies (21, 22, 24, 26). The information of the basic building block of high-affinity LMW-HP/HS obtained from exhaustive enzymatic digestion and nitrous acid degradation was input into Seq-GAG software. The pool containing predicted sequences with their calculated relative abundance was generated by Seq-GAG software.

### Model Building and Molecular Docking

Molecular docking of the interaction between the DIRAS2 protein and HP or HS decasaccharide was performed using AutoDock Vina software (The Scripps Research Institute). The structure of HP decasaccharide with 14 sulfate groups [ΔUA2S-GlcNS-IdoA2S-GlcNS6S-IdoA2S-GlcNS6S-IdoA2S-GlcNS6S-IdoA2S-GlcNS6S] and HS decasaccharide with 11 sulfate [IdoA2S-GlcNS6S-IdoA2S-GlcNS6S-IdoA2S-GlcNS6S-GlcA-GlcNS6S-GlcA-GlcNAc] were derived from the reported structure (Protein Data Bank [PDB] code: 1HPN). The structure of the DIRAS2 protein was derived from the reported structure of DIRAS2 protein complex (PDB code: 2ERX) and optimized by the CHARMM force field. All hydrogen atoms were added to the DIRAS2 protein and charged. A full-coverage box of size (47.25, 47.25, and 47.25) with a grid center (0.043, 18.032, and 19.453) was set up for docking. All monosaccharide rings in the decasaccharide sequences of HP and HS could rotate freely, and ring substitutes of carboxylate–sulfate groups were defined as flexible. The decasaccharide was allowed to move freely around the surface of the DIRAS2 in the box. The complex poses were ranked based on binding energy in kcal/mol, and the lowest energy one was chosen as the most stable complex for binding site analysis. Analysis is carried out using the docking model using PyMOL 2.5.5 software (DeLano Scientific LLC).

## Result and Discussion

### Structural Characterization of HP and HS

**Fig. 1:**
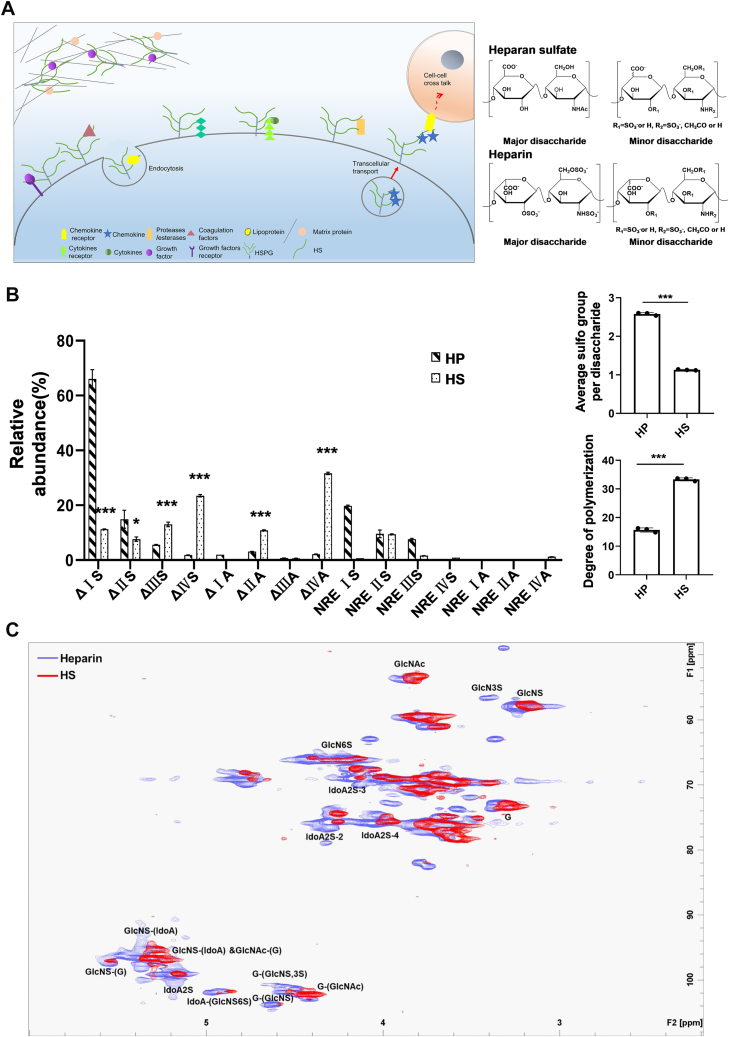
**Basic structural characterization of HP and HS.***A*, location and structural features of HP–HS. *B*, tructural characterization of HP and HS by LC–MS/MS MRM. *C*, ^1^H–^13^C HSQC NMR. Notes: ΔIVA, ΔUA-GlcNAc; ΔIIIA, ΔUA2S-GlcNAc; ΔIIA, ΔUA-GlcNAc6S; ΔIA, ΔUA2S-GlcNAc6S; ΔIVS, ΔUA-GlcNS; ΔIIIS, ΔUA2S-GlcNS; ΔIIS, ΔUA-GlcNS6S; ΔIS, ΔUA2S-GlcNS6S; NRE, nonreducing end. Data statistic analysis: ∗*p* < 0.05; ∗∗*p* < 0.01; and ∗∗∗*p* < 0.001 (mean ± SD, n = 3). HP, heparin; HS, heparin sulfate; HSQC, heteronuclear single quantum coherence; MRM, multiple reaction monitoring.

The structural features of HP and HS were characterized by LC–MS/MS MRM and ^1^H–^13^C HSQC NMR, with the NMR applied only for qualitative analysis. The detailed parameters of LC–MS/MS MRM are listed in Table S1. Both HP and HS consisted of eight disaccharide units, each exhibiting distinct sulfation or acetylation substitution patterns. Quantitative analysis of the unsaturated disaccharides and the saturated disaccharides released from the polysaccharide backbone was quantified using LC–MS/MS MRM, which provided the Mw and sulfation degree calculations for HP and HS. As shown in Figure 1*B*, ΔIS (ΔUA2S-GlcNS6S) was the most abundant disaccharide in HP, whereas ΔIVA (ΔUA-GlcNAc) predominated in HS. The relative abundance of ΔIS was 67.3% in HP compared with 11.3% in HS (*p* < 0.001), whereas ΔIVA accounted for 2.2% in HP and 30.0% in HS (*p* < 0.001). In addition, four disaccharides showed significant differences between HP and HS. The content of ΔIIS (ΔUA-GlcNS6S) was significantly higher in HP than in HS (*p* < 0.05), whereas the contents of ΔIIIS (ΔUA2S-GlcNS), ΔIVS (ΔUA-GlcNS), and ΔIIA (ΔUA-GlcNAc6S) were significantly lower in HP compared with HS (*p* < 0.001). The degree of sulfation, calculated from the disaccharide composition, also differed significantly (*p* < 0.001), with HP containing an average of 2.5 sulfate groups per disaccharide and HS containing 1.1 (Fig. 1*B*, *right*). Similarly, the Mw of HP (15.6 kDa) was significantly lower than that of HS (33.3 kDa) (*p* < 0.001) (Fig. 1*B*, *right*). In the ^1^H–^13^C HSQC NMR analysis (Fig. 1*C*), the signal assignments for the monosaccharide compositions of HP and HS also supported the aforementioned conclusion that although HP and HS share the same basic building blocks, the proportions of the eight disaccharides and the degree of sulfation differ significantly. The characterization of these structural differences forms the basis for a comprehensive understanding of the differences in bioactivity between HP and HS.

### Diversity of Anticancer and Anti-Injury Activities of HP and HS in the Liver by the Cellular Experiment

**Fig. 2:**
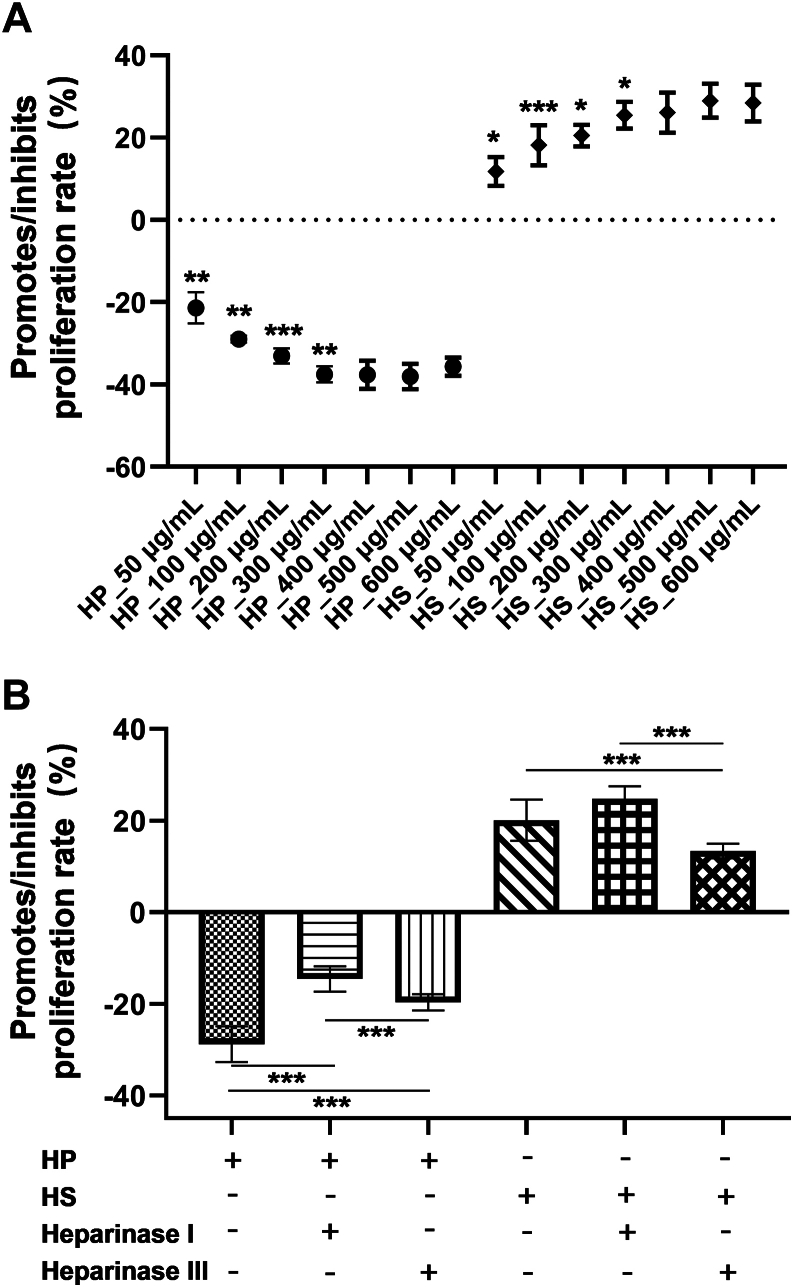
**HP and HS treatments for regulating the proliferation of HepG2 cells.***A*, different concentrations of HP and HS treatments on HepG2 cell proliferation. *B*, differential effects of heparinase I/III-treated HP or HS on HepG2 cell proliferation. Data statistic analysis: ∗*p* < 0.05; ∗∗*p* < 0.01; ∗∗∗*p* < 0.001 (mean ± SD, n = 6). HP, heparin; HS, heparin sulfate.

The results of the effects of HP and HS on HepG2 cells showed a distinct difference in promoting cell proliferation, which was recognized as a cellular basis for anticancer and anti-injury activities in the liver. HP exhibited significant inhibitory effects on HepG2 cell proliferation, whereas HS demonstrated the opposite trend. As shown in Figure 2*A*, the inhibition or promotion induced by HP or HS reached its highest point at a concentration of 300 μg/ml, with 37.5% inhibition and 24.5% promotion, respectively. Although a slight concentration-dependent trend appeared at 400 to 600 μg/ml, no significant difference was observed compared with 300 μg/ml; therefore, 300 μg/ml was selected for subsequent studies.

A partial enzymatic digestion method for HP and HS was then used to investigate why their structures, though similar, show significant differences in cell proliferation activity (Fig. 2*B*). Heparinase I and heparinase III were used to specifically digest the highly sulfated and nonsulfated regions of HP and HS, respectively, with heparinase I primarily hydrolyzing the highly sulfated region containing the NS domain and heparinase III primarily hydrolyzing the low-sulfated region containing the NAc domain. HP exhibited a significant decrease in its inhibitory effect on cell proliferation after treatment with either heparinase I or III, with the greatest reduction observed after treatment with heparinase I. This indicated that the highly sulfated structural domain of HP, represented by the ΔUA2S-GlcNS6S unit, played a crucial role in inhibiting cell proliferation. A significant decrease was also observed after treatment with heparinase III, suggesting that the structural domain represented by ΔUA-GlcNAc6S also contributed importantly to the inhibition of cell proliferation. In HS, by contrast, there was a slight but not significant increase in cell proliferation–promoting activity after heparinase I treatment, whereas a significant decrease was observed after heparinase III treatment. This suggested that the NS structural domain in HS played a limited role in cell proliferation compared with the NAc structural domain. According to the MRM results, HS contained a higher number of NAc disaccharide units than HP, such as ΔUA-GlcNAc6S and ΔUA-GlcNAc, and a lower number of highly sulfated NS disaccharide units, such as ΔUA2S-GlcNS6S. Consistent with our findings, Liu *et al.* prepared oligosaccharide mixtures capable of promoting or inhibiting tumor cell proliferation using heparinases I and III, respectively. The growth-promoting oligosaccharides contained more monosulfated and nonsulfated disaccharide fractions, whereas those with tumor proliferation–inhibiting activity contained more disulfated and trisulfated disaccharide units (17). Based on the combined results of cell proliferation assays, partial enzymatic digestion, and MRM analysis, it could be concluded that the highly sulfated structural domain of HP and the acetylated structural domain of HS played crucial roles in inhibiting and promoting cell proliferation, respectively.

### Label-Free Proteomics Analysis of HepG2 Cells Under HP and HS Treatments

**Fig. 3:**
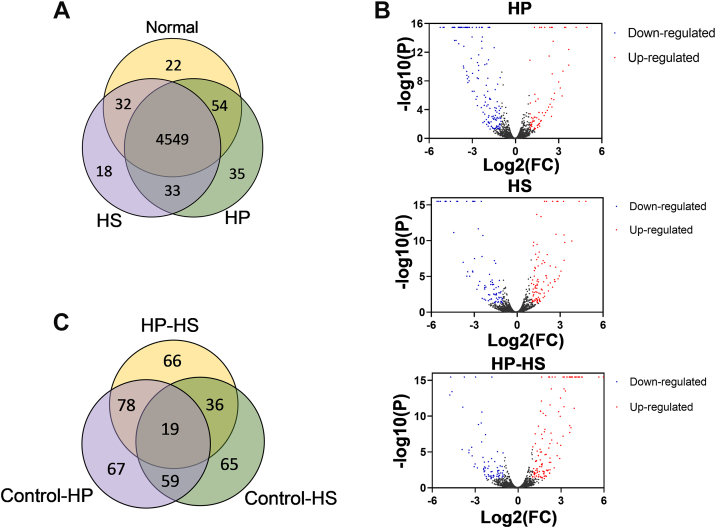
**Label-free quantitative proteomics identification for analyzing differential protein expression.***A*, Venn diagram of all identified proteins. *B*, Volcano plot of fold change among control group, HP, and HS. *C*, Venn diagram of identified differential proteins with upregulating/downregulating. HP, heparin; HS, heparin sulfate.

HP and HS exert biological functions by interacting with proteins. Therefore, characterizing the perturbation of protein expression profiles in hepatocytes by HP and HS at the proteomic level, analyzing changes in known and potential HS-binding proteins (HSBPs), and screening for key HSBPs may provide assistance in revealing the functional differences between HP and HS in various liver diseases. Label-free quantitative proteomics was employed to analyze the changes in the proteomic profiles of hepatocytes following HP and HS treatment. HepG2 cells were selected as an ideal model because of their stable state and high similarity compared with those in primary hepatocytes (27, 28). The proteomics experiment included three groups: control, HP, and HS, with three biological replicates per group. A total of 4743 proteins were identified across the three groups, among which 4549 were shared components (Fig. 3*A*). Compared with the control group, the HP and HS groups exhibited varying degrees of change greater than two or less than 0.5 and *p* < 0.05.

As shown in Figure 3, *B* and *C*, compared with the control group, 77 upregulated proteins and 146 downregulated proteins were identified in the HP group, whereas 98 upregulated and 81 downregulated proteins were identified in the HS group. Compared with the HP group, 125 upregulated proteins and 74 downregulated proteins were identified in the HS group. Among these, 19 differential proteins were shared, but their expression trends and fold changes differed between the HP and HS groups, indicating that HP and HS may exert distinct biological functions by differentially influencing protein expression within the same signaling pathways. Raw library search results for the proteomic data are available in the Supporting Information—Library Search Results and from the ProteomeXchange Consortium (https://proteomecentral.proteomexchange.org) under dataset identifier PXD064406.

### Functional Enrichment, Western Blotting Measurement, and Interaction Analysis of Differential Proteins

**Fig. 4:**
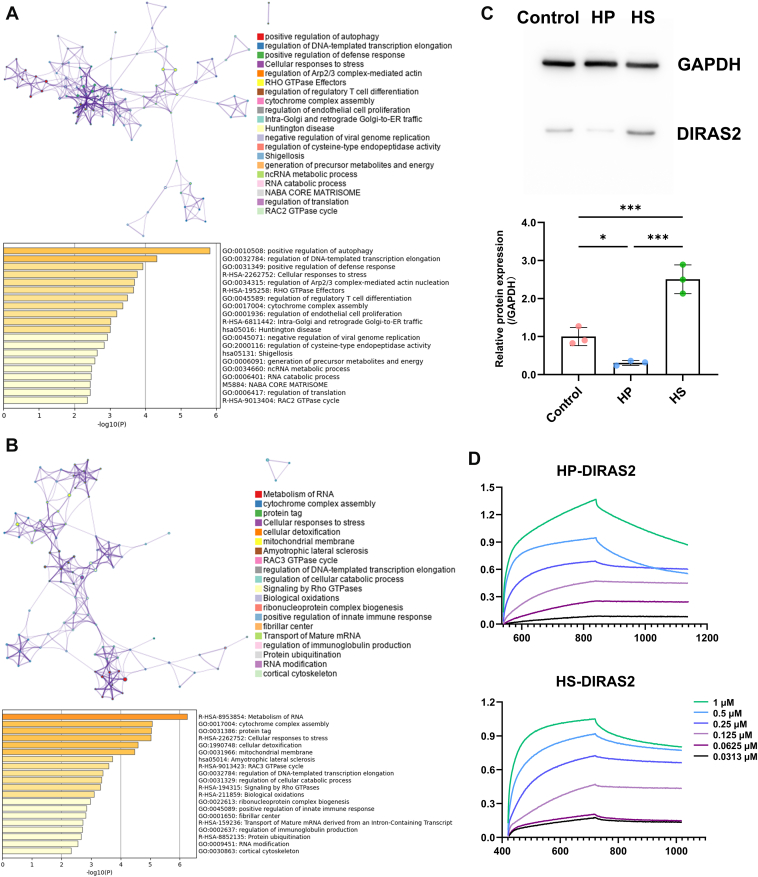
**Functional enrichment and interaction analysis of differential proteins.***A*, functional enrichment and interaction network of differential proteins in HP group. *B*, functional enrichment and interaction network of differential proteins in HS group. *C*, Western blotting measurement of DIRAS2 expression in control, HP, and HS groups. *D*, BLI characterization of HP and HS interactions with DIRAS2 protein. Data statistic analysis: ∗*p* < 0.05; ∗∗*p* < 0.01; ∗∗∗*p* < 0.001 (mean ± SD, n = 3). BLI, biolayer interferometry; DIRAS2, DIRAS family GTPase 2; HP, heparin; HS, heparin sulfate.

To explore the molecular functions and biological processes associated with proteomic perturbations in HepG2 cells caused by HP and HS, GO analysis was used to conduct functional enrichment analysis of the differential proteins in the HP group (Fig. 4*A*) and HS group (Fig. 4*B*), compared with the control group. Subsequently, the STRING database, Cytoscape software, and the MCODE plugin were used to analyze the interactions among these differential proteins. Raw results of the GO analysis are available in the Supporting Information—GO Analysis.

In the GO network, the size of each node was proportional to the number of genes involved. Different colors represented distinct functions or processes, and nodes with similar scores were connected by lines. The accompanying bar indicated the *p* value corresponding to each GO term. A total of 131 GO terms were enriched in the HP group, with the top 20 shown in Figure 4*A*. The results indicated that the differential proteins in the HP group were involved in various biological processes, including autophagy, DNA transcription, RHO GTPase signal transduction, regulation of cell proliferation, RAC2 GTPase cycle, and so on. A total of 122 GO terms were enriched in the HS group, with the top 20 shown in Figure 4*B*. Similarly, these enriched pathways included cellular detoxification, the RAC3 GTPase cycle, DNA transcription, RHO GTPase effectors, protein ubiquitylation, regulation of cell proliferation, and so on. Notably, within the shared pathways, RHO GTPase, RAC2, and RAC3 all belong to the GTPase superfamily, which was associated with various biological functions, including cell proliferation, cell polarity, and vesicle transport, as well as tumor growth, metastatic spread, and invasive potential (29, 30, 31). These findings suggested that cell proliferation, through interference with GTPase-related pathways, might be differentially modulated by HP and HS, thereby contributing to their distinct functional roles in liver cancer and liver injury.

The results of the major interaction network analysis are shown in Figures 4, *A*, *B*, S2, and S4, where blue and red nodes represented downregulated and upregulated proteins, respectively, and the size of the nodes was proportional to the frequency of protein–protein interactions. The complete interaction networks of all differential proteins are shown in Figs. S1 and S3 in the Supporting Information. The protein interaction subnetwork in the HP group consisted of 26 proteins, including three HSBPs: midkine (MDK), collagen alpha-1 chain of XVIII (COL18A1), and perlecan (HSPG2). MDK, COL18A1, and HSPG2 are all closely associated with tumorigenesis, progression, and prognosis. MDK is an HP-binding growth factor positively correlated with cell proliferation (32, 33). COL18A1 regulates cell proliferation and angiogenesis and performs multiple functions (34). High expression of HSPG2 had been associated with poor tumor prognosis (35). The protein interaction subnetwork in the HS group consisted of 35 proteins, including five HSBPs: microtubule-associated protein tau, lactotransferrin (LTF), vitamin D–binding protein (GC), complement component C9 (C9), ceruloplasmin (CP), and large ribosomal subunit protein eL29 (RPL29). Among these, LTF and RPL29 had been reported to promote cell proliferation through overexpression (36, 37). Among the differences in biological activity, proteins exhibiting significant expression changes but in opposite directions were of particular interest. In the two subnetworks, several such proteins were identified, including DIRAS2, C9, TUBB8, APOA4, and CRIP1. Notably, HP downregulated DIRAS2 abundance in HepG2 cells, whereas HS upregulated it. DIRAS2, a member of the GTPase family (38), could regulate cell proliferation by activating the RAS–mitogen-activated protein kinase signaling pathway (39, 40) and might play a key role in modulating proliferation under HP and HS intervention. Therefore, DIRAS2 was selected as the target protein for subsequent studies.

Given that label-free quantitative proteomics provided a relatively simple but less accurate method for protein quantification, Western blotting of target protein was employed to quantify the relative expression of DIRAS2 protein in the cellular model. As shown in Figure 4*C*, HP treatment significantly downregulated the expression of DIRAS2 in HepG2 cells compared with the control group (*p* < 0.05), whereas HS upregulated it (*p* < 0.001). The expression change of DIRAS2 in HepG2 cell confirmed by Western blotting was entirely consistent with the trend in label-free quantitative proteomics, confirming the conclusion that the protein exhibited an opposite trend of change in HP/HS-treated HepG2 cells. The raw figures of Western blotting measurement of DIRAS2 are listed in the Supporting Information Fig. S5 to Fig.S7.

BLI was then used to assess the binding affinity of DIRAS2 with HP and HS, further confirming the interaction between DIRAS2 and these two polysaccharides (Fig. 4*D*). Bovine serum albumin, as a classical protein that does not interact with HP and HS, was used as a negative control to demonstrate that the BLI method exhibited no nonspecific adsorption (shown in Figs. S8 and S9). The results showed that DIRAS2 had strong binding affinities with both HP and HS, with *K*_*D*_ of 11.01 ± 0.224 nM and 18.96 ± 0.376 nM, respectively. These findings indicated that DIRAS2, as a newly identified HP–HSBP, might play a crucial role in the regulation of cell proliferation and represents a promising target for *in vivo* validation in animal models that more closely mimic the *in vivo* liver environment. In addition, microtubule-associated protein tau, LTF, GC, C9, CP, RPL29, MDK, COL18A1, and HSPG2 might also be key HSBPs involved in this regulatory network.

### Functional Diversity Verification of HP–HS by Animal Experiment and Immunohistochemical Analysis

**Fig. 5:**
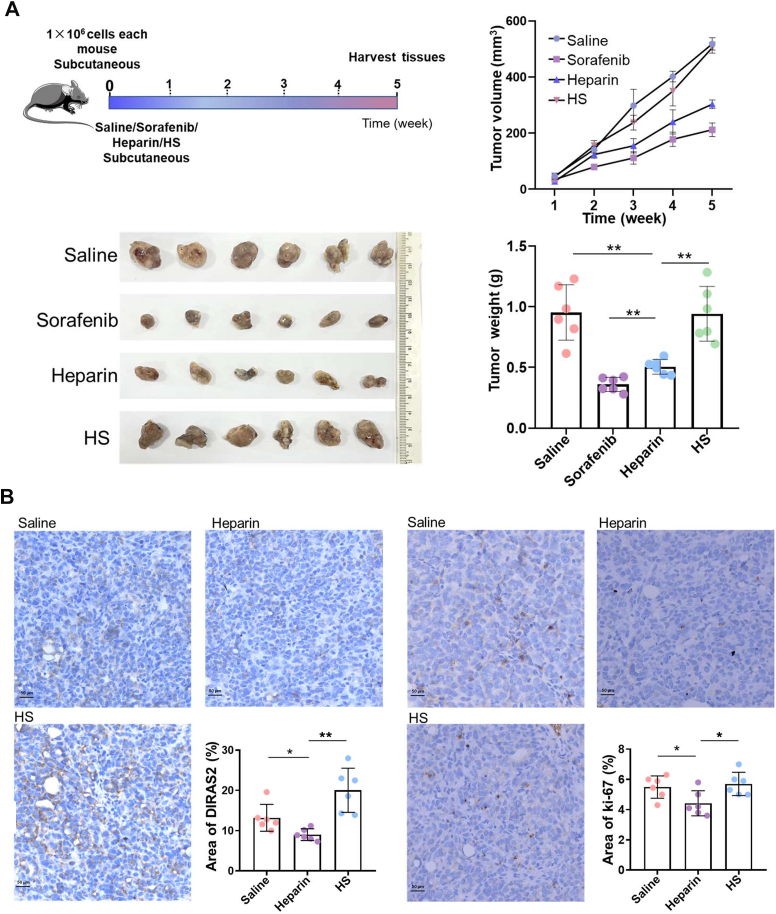
**Animal experimental procedure and immunohistochemical validation of key proteins in the liver cancer model.***A*, experimental procedure of the liver cancer model in mice. *B*, immunohistochemical validation of key proteins in liver cancer model. Data statistic analysis: ∗*p* < 0.05; ∗∗*p* < 0.01; ∗∗∗*p* < 0.001 (mean ± SD, n = 6).

**Fig. 6:**
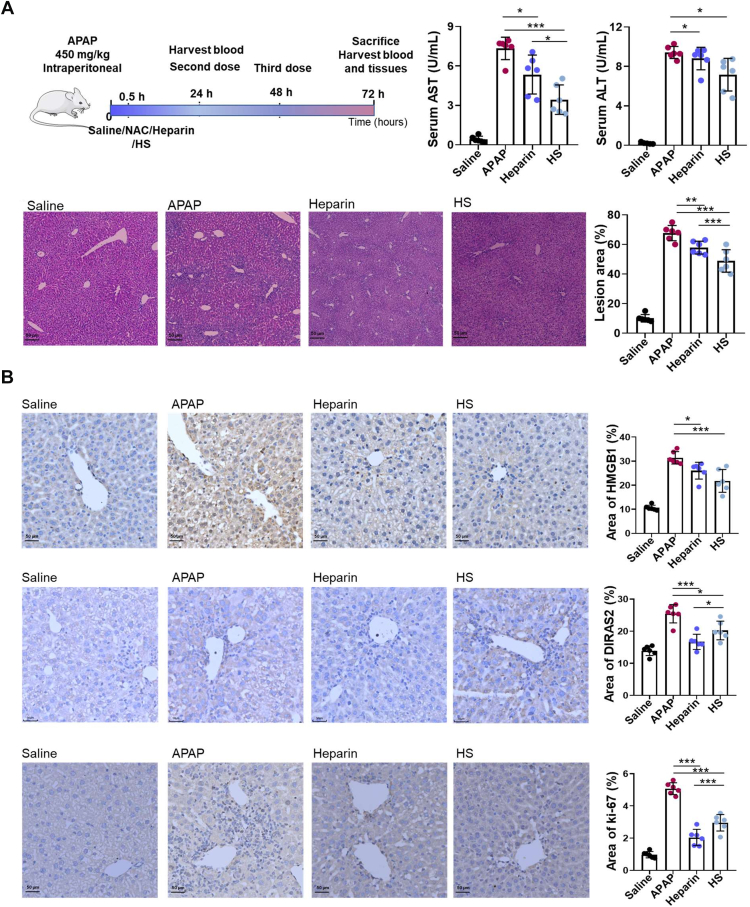
**Animal experimental procedure and immunohistochemical validation of key proteins in the APAP-induced liver injury model.***A*, experimental procedure of APAP-induced liver injury in mice. *B*, immunohistochemical validation of key proteins in the APAP-induced liver injury model. Data statistic analysis: ∗*p* < 0.05; ∗∗*p* < 0.01; ∗∗∗*p* < 0.001 (mean ± SD, n = 6). APAP, acetaminophen.

The regulatory effects of HP and HS on cell proliferation and their therapeutic potential in liver cancer and liver injury were validated through animal experiments, and the results were fully consistent with those of the cellular assays. In the tumor model established by HepG2 cell injection, HP significantly inhibited tumor growth compared with the control group (*p* < 0.01). At 5 weeks, tumor volume in the HP group was 40% smaller, and tumor weight was 47% lower than in the model group (Fig. 5*A*). No significant differences in tumor volume or weight were observed in the HS group. These results indicated that HP exhibits strong antitumor activity in both *in vitro* and *in vivo* models, whereas HS showed no significant inhibitory effect, suggesting that HP has greater potential for combating liver cancer than HS. In the APAP-induced liver injury model, although both HP and HS reduced liver injury area, HS led to a more substantial decrease in serum aminotransferase concentrations than HP (*p* < 0.05). Compared with the control group, the injury area was reduced by 14% in the HP group (*p* < 0.01) and by 28% in the HS group (*p* < 0.001) (Fig. 6*A*). These results demonstrated that HS has stronger antiliver injury activity than HP.

To further verify whether the differences in the bioactivities of HP and HS in liver cancer and liver injury were related to the DIRAS2 protein, the distribution and abundance of DIRAS2 in tumor and liver-injured tissues were examined using immunohistochemical techniques (shown in Figs. 5 and 6). In the liver cancer model, the DIRAS2-positive area in tumor tissue was significantly reduced in the HP group compared with the APAP group (*p* < 0.05), whereas the DIRAS2-positive area was significantly increased in the HS group (*p* < 0.01) (Fig. 5*B*). The expression of Ki-67, a nuclear protein positively associated with cell proliferation, was also assessed. Compared with the control group, the percentage of Ki-67-positive cells in the HP group was significantly lower (*p* < 0.05), decreasing from 5.5% to 4.3%, whereas the percentage in the HS group did not change significantly but differed markedly from the HP group (*p* < 0.05). The distributions and abundances of HMGB1 (an inflammatory factor positively associated with liver injury) (15), DIRAS2, and Ki-67 were further evaluated in liver-injured tissues (Fig. 6*B*). The results showed that the HMGB1-positive area was significantly lower in both the HP and HS groups (*p* < 0.001). However, the DIRAS2- and Ki-67-positive areas were significantly higher in the HS group than in the HP group, measuring 18.6% and 3.2% in the HS group *versus* 13.51% and 2.04% in the HP group (*p* < 0.05 and *p* < 0.001, respectively). Notably, the positive areas of DIRAS2 and Ki-67 in both treatment groups were smaller than those observed in the APAP group, which might be attributed to endogenous cell proliferation triggered as part of tissue repair in response to severe damage. In addition, the data for sorafenib and NAC were not discussed, presented, or evaluated because of their identical effects as previously reported (7, 15), and for reasons of simplicity.

In general, the aforementioned results indicated that HP could reduce the intracellular distribution of DIRAS2 and decrease the proliferation rate of tumor cells, whereas HS did not exhibit this biological activity. This might be one of the mechanisms by which HP inhibited tumor cell proliferation and exerted its antitumor effects. Both HP and HS demonstrated antihepatic injury activity by neutralizing HMGB1, consistent with previous findings (15). Moreover, HS was able to promote hepatocyte proliferation by upregulating DIRAS2 expression and thereby contributed to liver tissue regeneration following injury, in contrast to HP. It should be further clarified that DIRAS2 protein, being highly correlated with cell proliferation, exhibited consistent expression changes in both cell and animal models. In contrast, HMGB1 protein, as an inflammatory factor, was only upregulated during inflammatory responses. As a preliminary screening and investigation of cell proliferation under HP–HS administration, we did not employ an inflammatory cell model. Although HP and HS could alter cellular proliferation states, they did not induce inflammatory reactions. Consequently, HMGB1 protein showed no expression alterations in the cellular proteomics analysis.

Thus, HS exhibited superior activity against DILI compared with HP, with a lower risk of internal bleeding. Previous studies had shown that HP exerted antitumor effects by inhibiting angiogenic factors and heparanase (41, 42). Our results further suggested that HP might also suppress tumor cell proliferation by modulating intrinsic signaling pathways within tumor cells.

### Structural Features of High-Affinity HP and HS Oligosaccharide

In the preceding experiments, our results demonstrated that DIRAS2 is an HSBP differentially modulated by HP and HS. Considering that HP and HS exert their biological effects *via* protein interactions, it is likely that DIRAS2 engages in different binding modes or mechanisms with each polysaccharide. Therefore, the precise structures of high-affinity HP/HS oligosaccharides were investigated using targeted protein-affinity chromatography and cluster sequencing strategies to elucidate the distinct binding mechanisms underlying HP/HS–DIRAS2 interactions.

**Fig. 7:**
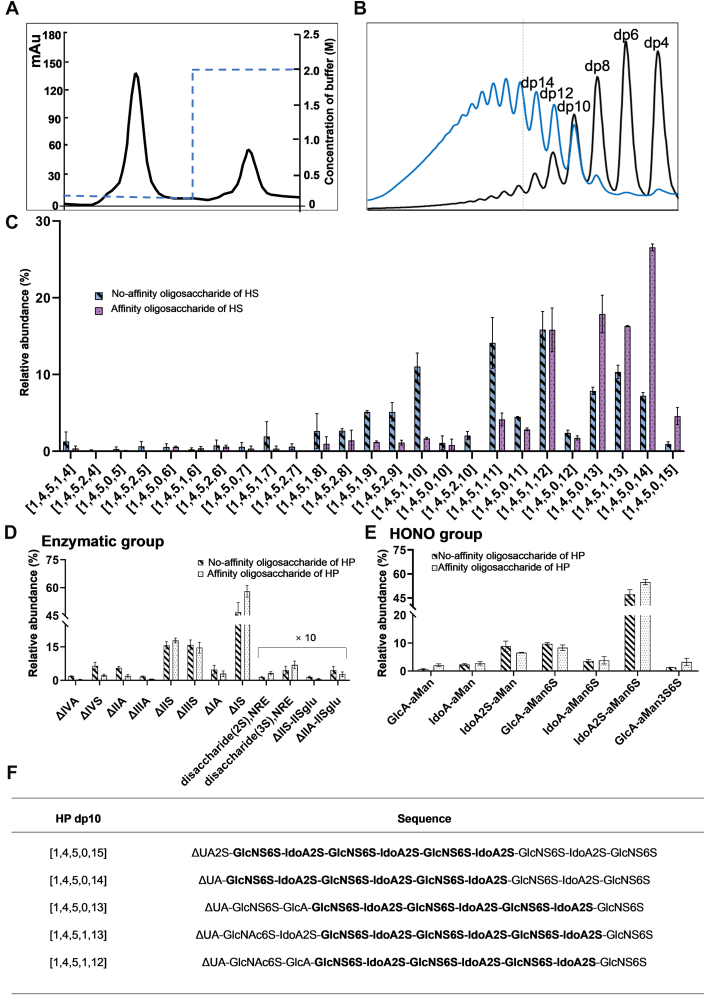
**Cluster sequencing strategy of HP affinity decasaccharide.***A*, affinity chromatography of HP oligosaccharide. *B*, the degree of polymerization analysis of the HP affinity oligosaccharide. *C*, HILIC–MS of HP affinity-dp10. *D*, the enzymatic group of HP affinity-dp10. *E*, HONO group of HP affinity-dp10. *F*, predicted sequences of representative HP affinity-dp10 components (top 5). HLIC, hydrophilic interaction liquid chromatography; HP, heparin; MS, mass spectrometry.

**Fig. 8:**
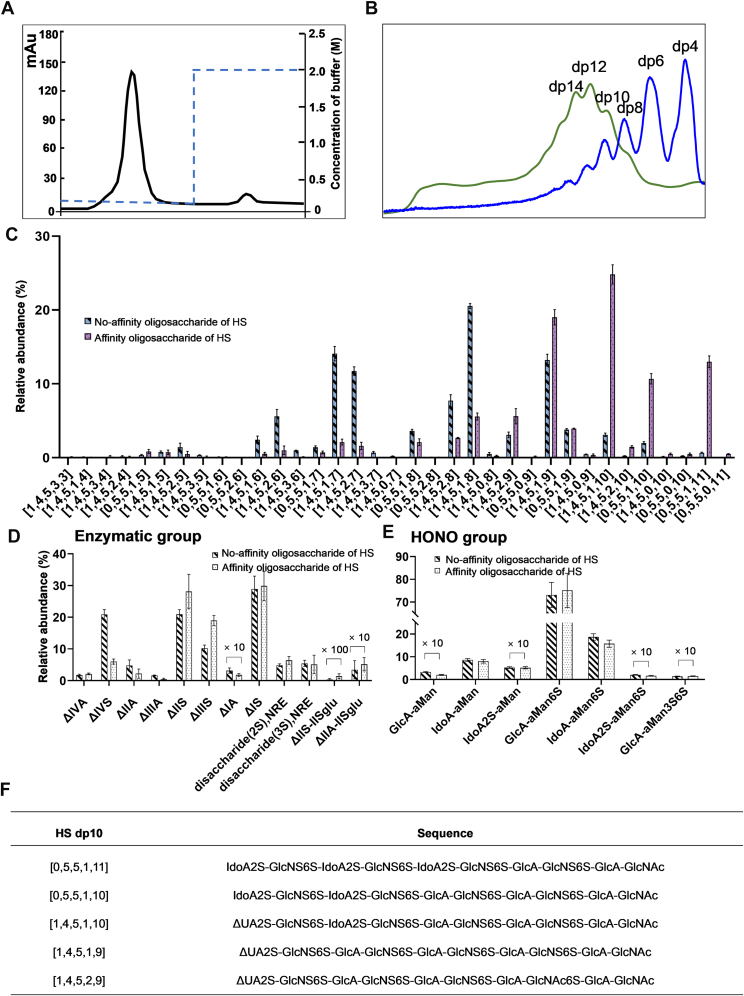
**Cluster sequencing strategy of HS affinity decasaccharide.***A*, affinity chromatography of HS oligosaccharide. *B*, the degree of polymerization analysis of the HS affinity oligosaccharide. *C*, HILIC–MS of HS affinity-dp10. *D*, the enzymatic group of HS affinity-dp10. *E*, HONO group of HS affinity-dp10. *F*, predicted sequences of representative HS affinity-dp10 components (top 5). Notes: ΔIVA, ΔUA-GlcNAc; ΔIIIA, ΔUA2S-GlcNAc; ΔIIA, ΔUA-GlcNAc6S; ΔIA, ΔUA2S-GlcNAc6S; ΔIVS, ΔUA-GlcNS; ΔIIIS, ΔUA2S-GlcNS; ΔIIS, ΔUA-GlcNS6S; ΔIS, ΔUA2S-GlcNS6S; disaccharide (2S) NRE, IdoA-GlcNS6S; disaccharide(3S). NRE, IdoA2S-GlcNS6S; ΔIIS-IISglu, ΔUA-GlcNS6S-GlcA-GlcNS3S6S; ΔIIA-IISglu, ΔUA-GlcNAc6S-GlcA-GlcNS3S6S. HILIC, hydrophilic interaction liquid chromatography; HS, heparin sulfate; NRE, nonreducing end.

The results suggested that only a small portion of HP–HS oligosaccharides could bind to DIRAS2 (Figs. 7*A* and 8*A*), and the degree of polymerization distributions of the high-affinity oligosaccharides are shown in Figures 7*B* and 8*B*. Compared with the original oligosaccharide mixtures, the proportion of components with higher degrees of polymerization significantly increased in the high-affinity oligosaccharides. Notably, all components with significantly increased abundance in DIRAS2 high-affinity oligosaccharides from both HP and HS were dp10. Subsequently, HILIC–MS was used to qualitatively and relatively quantitatively analyze the original HP/HS oligosaccharides and the DIRAS2-binding components (Figs. 7*C* and 8*C*). A total of 25 HP decasaccharide components were identified. Among them, the relative abundance of the affinity oligosaccharide [1,4,5,0,15] increased significantly from 0.94% to 4.58%. This structure was assigned as a fully sulfated decasaccharide, which may provide maximal electrostatic interactions during protein binding. In addition, the relative abundances of other highly sulfated components, such as [1,4,5,0,14], [1,4,5,0,13], [1,4,5,1,13], and [1,4,5,1,12], also increased significantly, indicating that DIRAS2 exhibited some sequence selectivity for the HP chain. The high-affinity oligosaccharides of HS displayed a similar trend to those of HP, with significantly increased levels of sulfation and increased relative abundance of highly sulfated components. Interestingly, components containing saturated uronic acid, such as [0,5,5,1,10] and [0,5,5,1,11], also increased significantly (*p* < 0.001). These findings suggested that the highly sulfated components containing saturated uronic acid are located at the reducing end of the original HS chain, consistent with the domain architecture of natural HS. In addition, the relative abundances of components such as [1,4,5,1,10], [1,4,5,1,9], [0,5,5,1,10], [0,5,5,1,11], and [1,4,5,2,9] also increased significantly. The degree of sulfation in high-affinity HS oligosaccharides was notably lower than in those of HP, and at least one *N*-acetyl group was present in each major HS component. This structural feature might be closely related to the distinct biological activities of HS and HP.

The sequences of the high-affinity oligosaccharides were determined using the Seq-GAG software, which integrates the types and contents of basic components in the affinity oligosaccharides and generates predicted sequences for each component based on a theoretical database and structural characteristics (26). The basic components and their relative abundances, obtained through combined LC–MS analysis of complete enzymatic hydrolysis and nitrous acid degradation, were input into the Seq-GAG software. As a result, the predicted sequences of high-affinity HP and HS decasaccharides were generated (Figs. 7, *D*, *E* and 8, *D*, *E*). The sequencing results (Figs. 7*F* and 8*F*) indicated that three consecutive IdoA2S-GlcNS6S disaccharide units were essential components of the HP decasaccharide–binding domain. The variable regions of the HP-binding domain primarily included two disaccharide types: GlcA-GlcNS6S and GlcA-GlcNAc6S. In the HS decasaccharide–binding domain, one to three IdoA2S-GlcNS6S disaccharide units were also observed. However, the invariable region of the HS decasaccharide was shorter than that of HP, and a greater diversity of variable sequences was present, including GlcA-GlcNS6S, GlcA-GlcNAc6S, and GlcA-GlcNAc. These results indicated that DIRAS2 preferentially bound highly sulfated motifs in HP oligosaccharides, whereas HS oligosaccharides exhibited greater sequence variability. Components with higher sulfation carried a larger negative charge and possessed stronger binding affinity to target proteins. These highly sulfated components might competitively bind to DIRAS2, thereby influencing biological activity. Notably, the binding preferences of DIRAS2 for HS oligosaccharides differed from those for HP. The degree of sulfation in high-affinity HS oligosaccharides was significantly lower than in those of HP, and each major HS component contained at least one *N*-acetyl group at the reducing end. This structural feature was likely a key basis for the functional differences between HP and HS. Current drug development efforts based on the antitumor activity of HP primarily focus on unclassified HPs, including nonanticoagulant HP derivatives (43) and HP-based nanodelivery systems (44). However, there were relatively few reports on structurally defined saccharide-based drugs. Moreover, hepatic HSPG deficiency was strongly correlated with increased liver damage and inflammation (16), and exogenous HS enriched in *N*-sulfation and 2-*O*-sulfation had been shown to possess notable antiliver injury activity (17). Nevertheless, concerns remain regarding the safety of artificially synthesized HS. Altogether, these findings provided a structural basis for the rational design of targeted HP-derived antitumor candidate drugs and HS-derived therapeutics for liver injury.

### Molecular Mechanism of DIRAS2 and dp10-Affinity Interaction

**Fig. 9:**
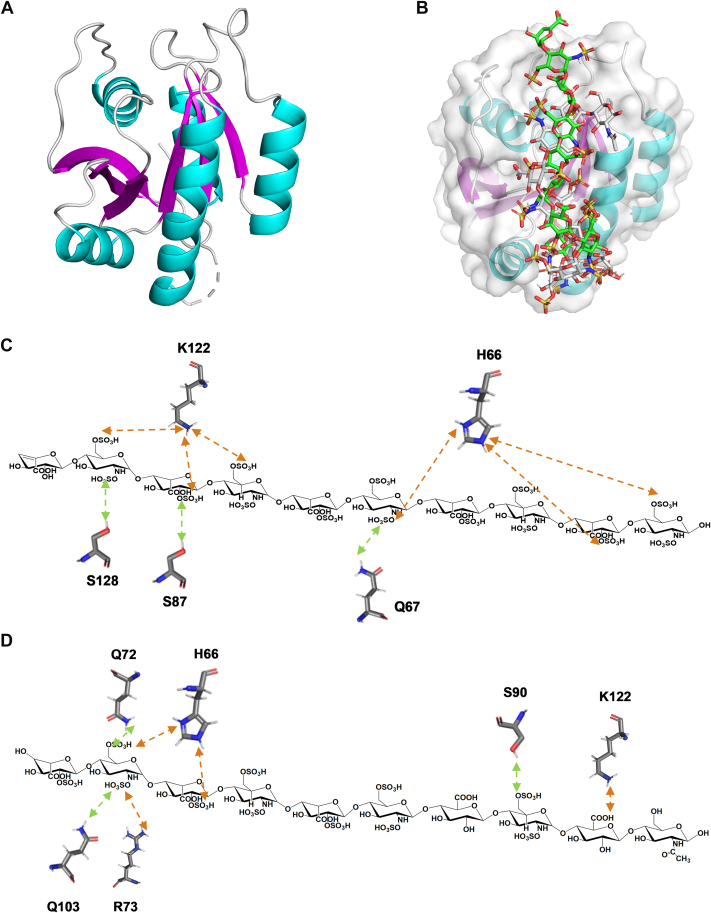
**Characterization of the binding sites involved in the interaction between HP–HS decasaccharide (derived from PDB:****1HPN****) and DIRAS2 protein (derived from PDB:****2ERX****) using molecular docking.***A*, the conformation of the DIRAS2 protein. *B*, the same binding region of DIRAS2 with HP and HS chains. *C*, the interactional contribution of DIRAS2 and HP binding motif. *D*, the interactional contribution of DIRAS2 and HS binding motif. Notes: *Orange* represented attractive charge, and *green* represented conventional hydrogen bond. DIRAS2, DIRAS family GTPase 2; HP, heparin; HS, heparan sulfate; PDB, Protein Data Bank.

AutoDock Vina software was used to simulate the binding complex formed between the DIRAS2 protein (derived from PDB code: 2ERX) and HP–HS decasaccharides. Binding poses were evaluated using predicted binding energies, and the conformation with the lowest energy was selected for further analysis. The best binding poses of the HP and HS decasaccharide–DIRAS2 complexes are shown in Figure 9, with binding affinities of −5.8 kcal/mol and −3.9 kcal/mol, respectively. As shown in Figure 9*B*, both HP and HS chains occupied the same binding site on the DIRAS2 protein, suggesting a potentially similar mode of interaction. Key amino acids such as His66 and Lys122 involved in decasaccharide binding are illustrated in Figure 9, *C* and *D*. The interactions between HP/HS decasaccharides and DIRAS2 were primarily mediated by electrostatic attractions formed between sulfate/carboxylate groups on the oligosaccharides and basic amino acid residues (Lys and His), with hydrogen bonding as a secondary interaction. The 2-*O*-sulfation, *N*-sulfation, 6-*O*-sulfation, and carboxyl groups on the decasaccharide chains contributed significantly to the interaction. The basic amino acid residues His66 and Lys122 in DIRAS2 played crucial roles in binding by forming electrostatic interactions. In addition, other residues, including Ser87, Ser90, Ser128, Gln67, and Gln72, further stabilized the complex through hydrogen bonding. In the HP binding model (Fig. 9*C*), His66 and Lys122 formed six electrostatic interactions with the 2-*O*-and-6-*O*-sulfation groups of the HP chain, whereas only three electrostatic interactions were observed in the HS chain (Fig. 9*D*), demonstrating that HP binds more strongly to DIRAS2 than HS. These results suggested that the sulfation pattern and three-dimensional conformation of the decasaccharide chains were critical determinants of binding specificity, and the differences in binding modes represented a molecular mechanism underlying the distinct biological activities of HP and HS.

## Conclusion

Our results demonstrated that the antiliver cancer activity of HP was superior to that of HS, whereas the ability of HS to promote tissue repair exceeded that of HP. DIRAS2, an HSBP closely associated with cell proliferation, was identified through label-free quantitative proteomics, bioinformatics analysis, BLI, and immunohistochemical techniques. Interestingly, DIRAS2 exhibited opposite expression trends in the HP and HS groups at both cellular and tissue levels. HP significantly reduced DIRAS2 abundance in tumor tissue and exerted an inhibitory effect on tumor cell proliferation. In contrast, both HP and HS neutralized the inflammatory factor HMGB1 and inhibited inflammation in the APAP-induced liver injury model. Moreover, HS promoted hepatocyte proliferation by increasing DIRAS2 expression in liver tissue. Cluster sequencing revealed that at least three consecutive GlcNS6S-IdoA2S units were required in the decasaccharide-binding domain of HP. In comparison, the HS-binding domain included IdoA2S-GlcNS6S, GlcA-GlcNS6S, and IdoA-GlcNAc structural units. Molecular docking simulations suggested that differences in binding modes between the HP–HS chains and the DIRAS2 protein were the key factors underlying their functional divergence.

## Data Availability

The mass spectrometry proteomics data had been deposited to the ProteomeXchange Consortium (https://proteomecentral.proteomexchange.org) *via* the iProX partner repository (45, 46) with the dataset identifier PXD064406. Other data files are stored at the National Glycoengineering Research Center, Shandong University, and are available from the corresponding author upon a reasonable request.

## Supplemental Data

This article contains supplemental data.

## Conflict of Interest

The authors declare no competing interests.
