# Supplementary material for: Structural Domain Analysis of Heparin and Heparan Sulfate Combined With Label-Free Quantitative Proteomics to Elucidate Their Functional Diversity in Liver Cancer and APAP-Induced Liver Injury
Source: Mol Cell Proteomics. 2025 Oct 14;24(11):101090. doi: 10.1016/j.mcpro.2025.101090 (PMC12639849; doi:10.1016/j.mcpro.2025.101090)
Supplement: Supplementary Figures [file mmc3.doc]

**Supporting Information**

**Table S1.** Component units of HP/HS and corresponding multiple reaction monitoring transitions.


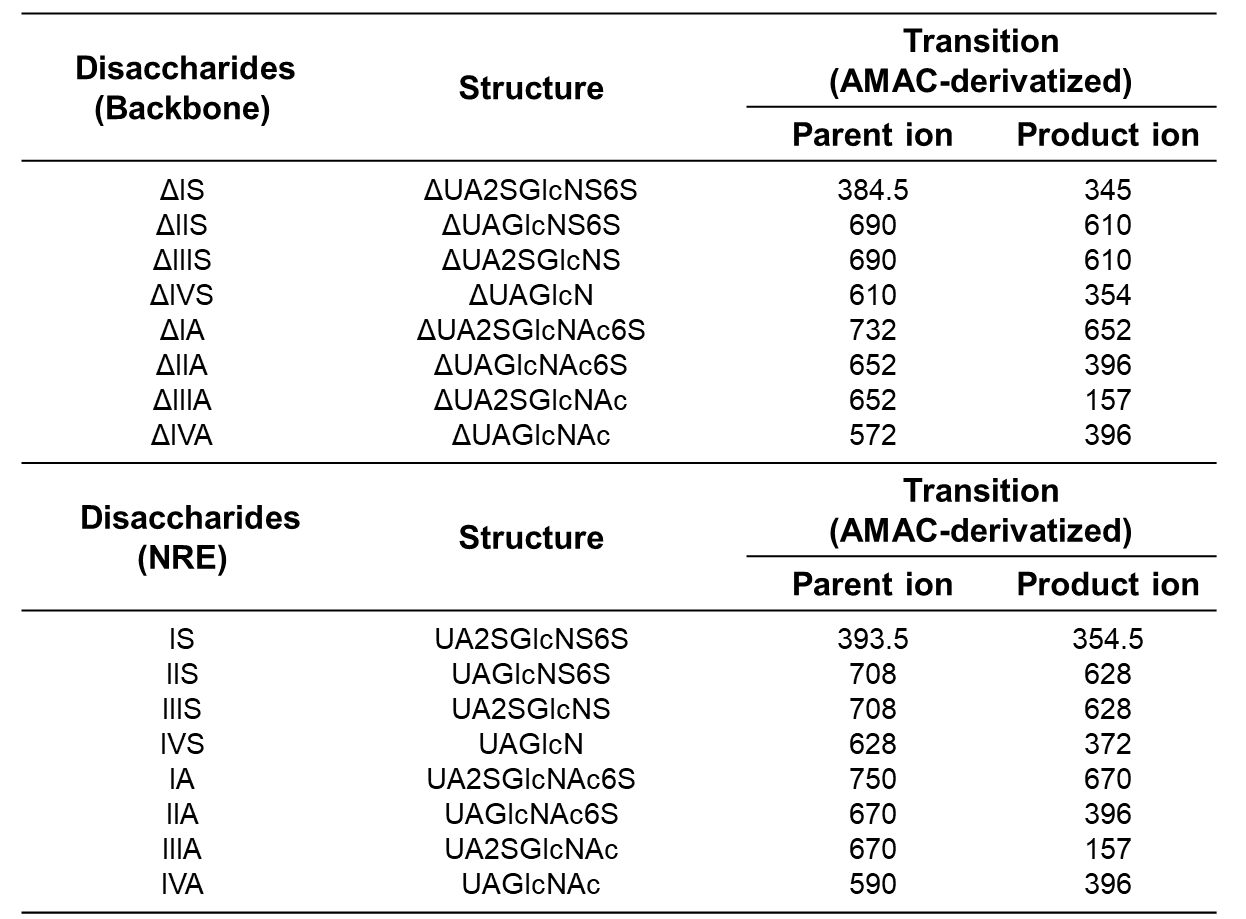


Notes: NRE represents non-reducing end. AMAC represents 2-Aminoacridone. UA represents uronic acid residue. GlcN represents N-glucosamine residue. ΔUA represents unsaturated uronic acid residues. 2S, 6S, NS, represents positions of sulfo group substitution. NAc represents *N*-acetylation substitution. Parent ion and Product ion represents the m/z values detected and quantified by LC-MS/MS MRM.


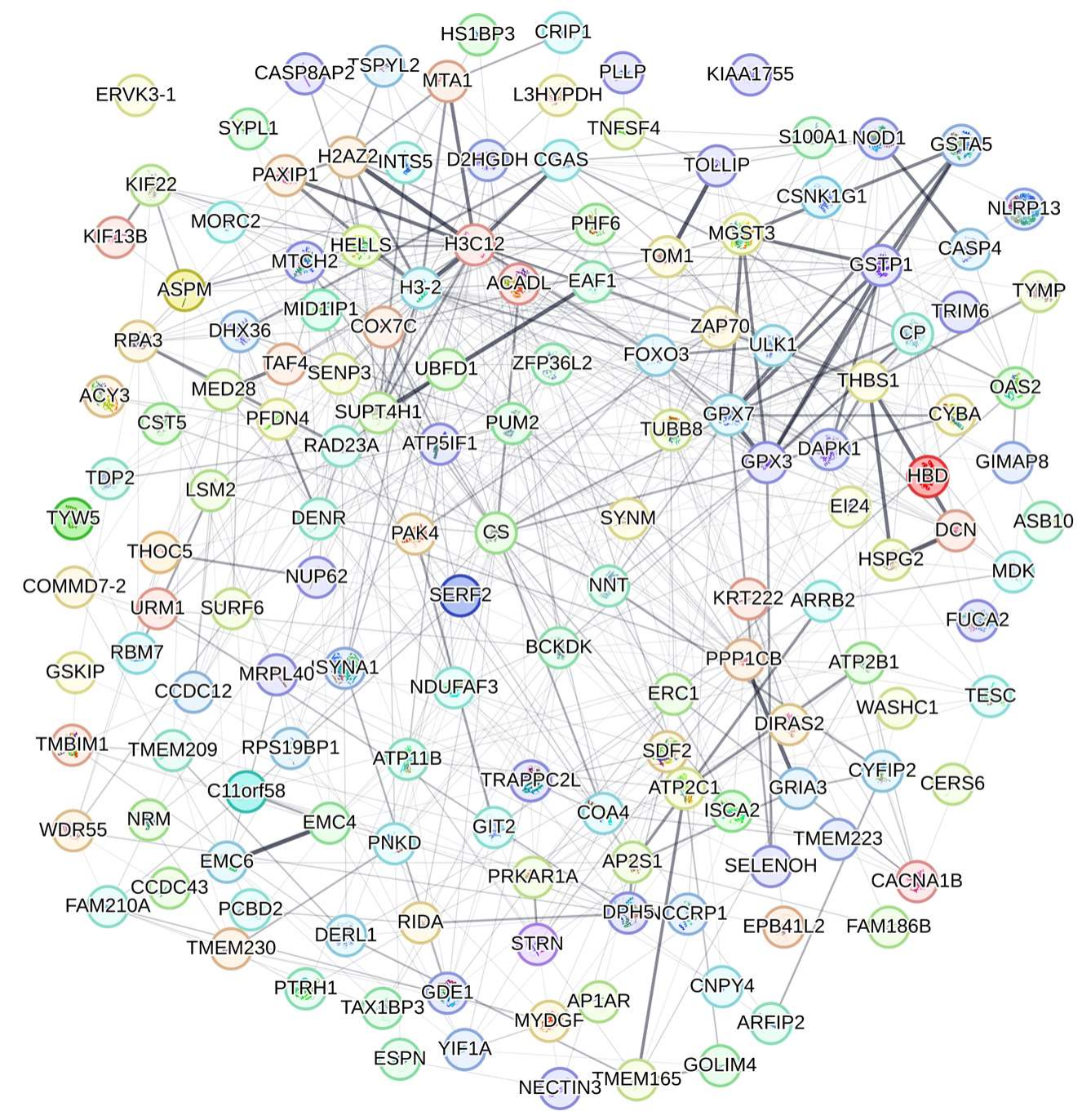


**Fig. S1.** Full interaction network of differential proteins in HP group.

**Fig. S2.** Interaction network of differential proteins in HP group with expressing change. Notes: Blue represents upregulation, red represents downregulation, and the intensity of the color represents the degree of expression.


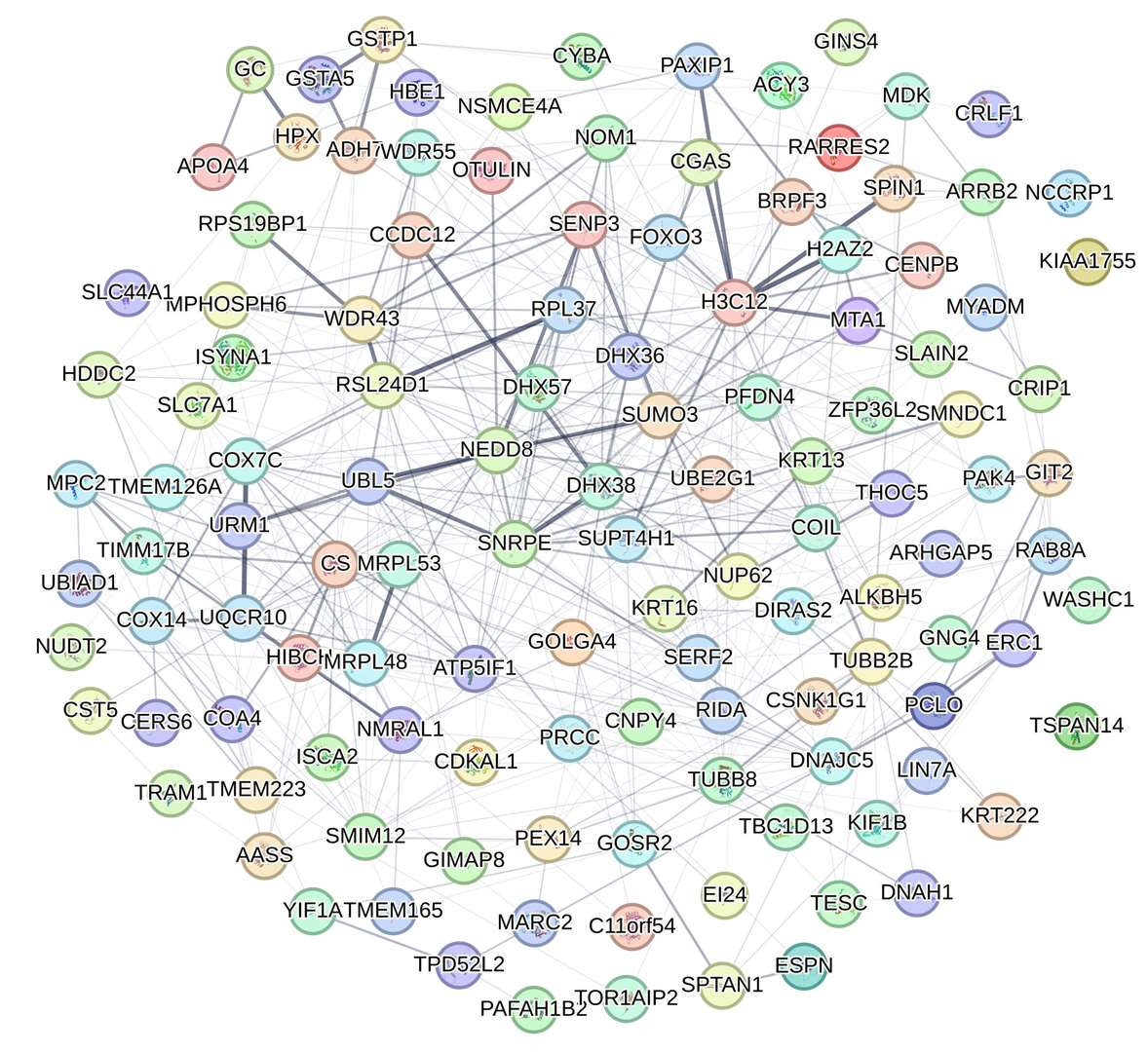


**Fig. S3.** Full interaction network of differential proteins in HS group.

**Fig. S4.** Interaction network of differential proteins in HS group with expressing change. Notes: Blue represents upregulation, red represents downregulation, and the intensity of the color represents the degree of expression.


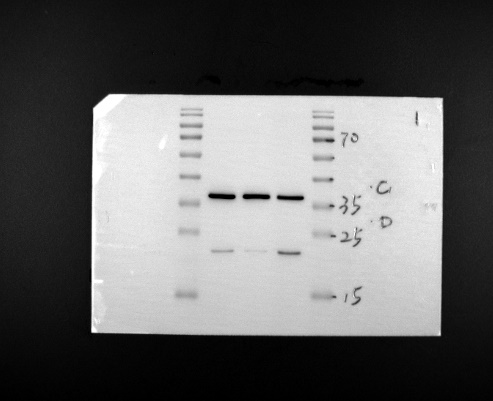


**Fig. S5.** Raw figure of western blotting measurement of DIRAS2 expression in control, HP and HS groups (parallel group 1).


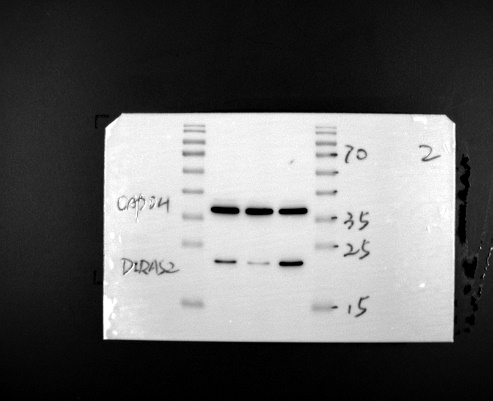


**Fig. S6.** Raw figure of western blotting measurement of DIRAS2 expression in control, HP and HS groups (parallel group 2).


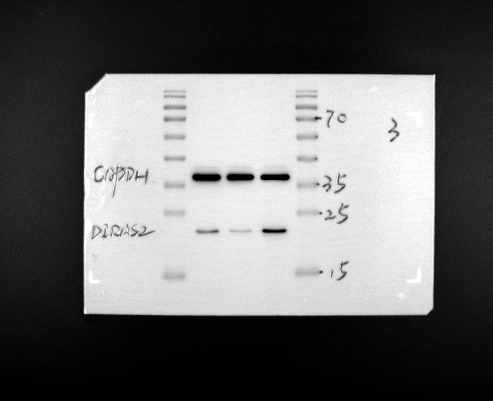


**Fig. S7.** Raw figure of western blotting measurement of DIRAS2 expression in control, HP and HS groups (parallel group 3).


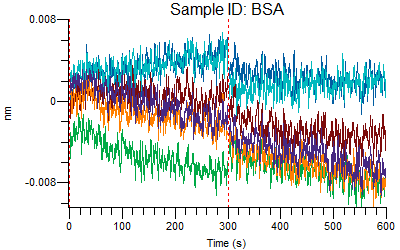


**Fig. S8.** BLI characterization of HP interaction with bovine serum albumin.


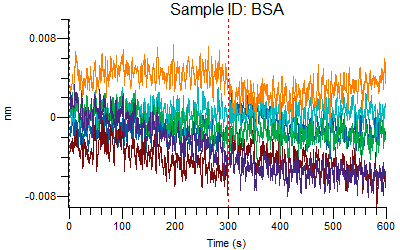


**Fig. S9.** BLI characterization of HS interaction with bovine serum albumin.


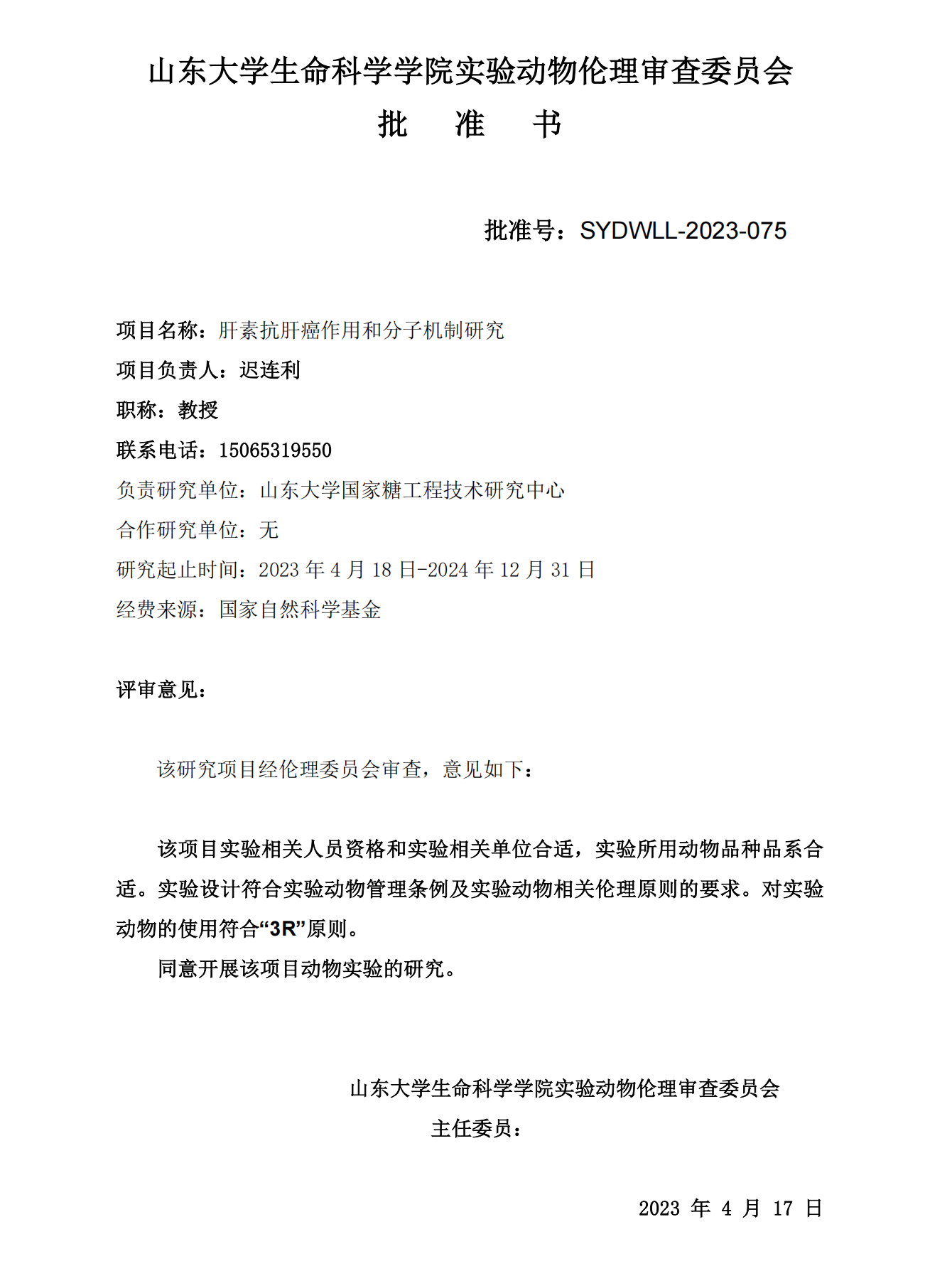


**Fig. S10.** Animal ethics approval letter of this study.


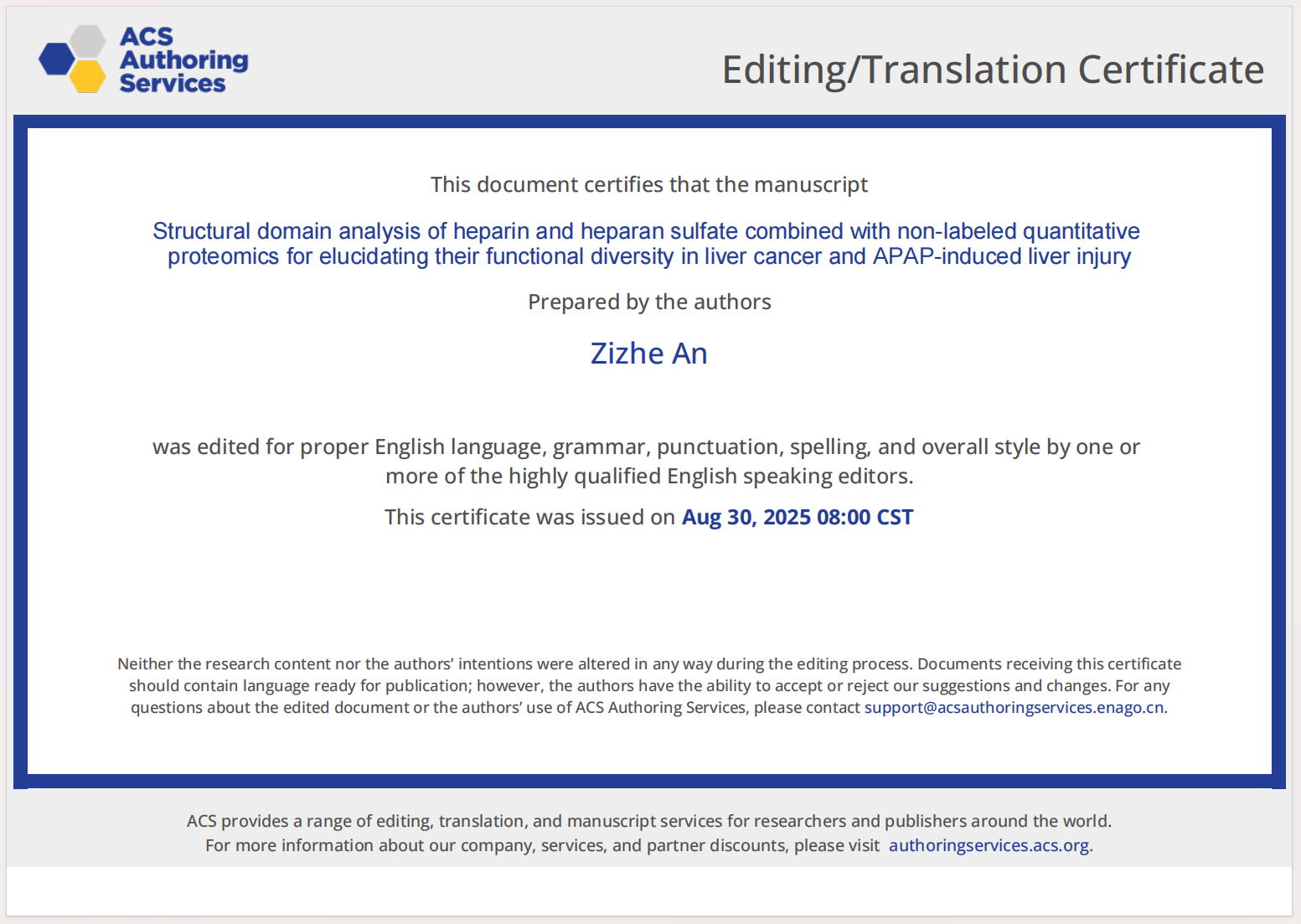


**Fig. S11.** Certificate of the language service.
